# Association of Serum Bilirubin With Metabolic Syndrome and Non-Alcoholic Fatty Liver Disease: A Systematic Review and Meta-Analysis

**DOI:** 10.3389/fendo.2022.869579

**Published:** 2022-07-20

**Authors:** Chen Liang, Zhiyuan Yu, Li Bai, Wei Hou, Shan Tang, Wei Zhang, Xinyue Chen, Zhongjie Hu, Zhongping Duan, Sujun Zheng

**Affiliations:** ^1^First Department of Liver Disease, Beijing You’an Hospital, Capital Medical University, Beijing, China; ^2^Beijing Municipal Key Laboratory of Liver Failure and Artificial Liver Treatment Research, Beijing You’an Hospital, Capital Medical University, Beijing, China; ^3^School of Medicine, Nankai University, Tianjin, China; ^4^Department of General Surgery, The First Medical Center, Chinese People's Liberation Army (PLA) General Hospital, Beijing, China; ^5^Fourth Department of Liver Disease, Beijing You’an Hospital, Capital Medical University, Beijing, China

**Keywords:** serum bilirubin, metabolic syndrome, non-alcoholic fatty liver disease, non-alcoholic steatohepatitis, meta-analysis

## Abstract

**Objective:**

Metabolic syndrome (MetS) and non-alcoholic fatty liver disease (NAFLD) are the leading chronic diseases worldwide. There are still many controversies about the association between serum bilirubin and MetS or NAFLD. This study aims to evaluate the association of serum total bilirubin (TBIL), direct bilirubin (DBIL), indirect bilirubin (IBIL) with MetS and NAFLD.

**Methods:**

Multiple databases were searched for relevant studies until November 2021. Randomized controlled trials, cross-sectional and cohort studies evaluating the association between serum bilirubin levels and MetS or NAFLD were included.

**Results:**

Twenty-four cross-sectional and cohort studies with 101, 517 participants were finally analyzed. Fifteen studies and 6 studies evaluated the association between bilirubin and MetS or NAFLD in health screening population, respectively, while 3 studies evaluated the association between bilirubin and non-alcoholic steatohepatitis (NASH) in NAFLD patients. Random effect model analysis showed the inverse association between TBIL and MetS in male (95%CI=0.71-0.96) and gender-neutral (95%CI=0.61-0.91) group. However, no significant association was found in females. Notably, the inverse association between DBIL and MetS was noticed in male (95%CI=0.36-0.75), female (95%CI=0.16-0.58) and gender-neutral population (95%CI=0.67-0.92). IBIL level was inversely associated with MetS in females (95%CI=0.52-0.96), whereas no statistical correlation presented in males. TBIL was not statistically correlated with NAFLD in gender-neutral or male subgroup. Similarly, there were no association between DBIL or IBIL and NAFLD in gender-neutral subgroup. However, the negative correlation between DBIL and NAFLD existed in males (95%CI=0.76-0.96). In NAFLD patients, IBIL analysis showed an inverse association with NASH (95%CI=0.01-0.12).

**Conclusion:**

Serum TBIL and DBIL levels, especially DBIL levels, assume an inverse correlation with MetS in healthy population. Serum IBIL is inversely associated with the onset and degree of NASH in NAFLD patients. Exogenous bilirubin supplement may be a potential strategy to assist in lowering the risk of developing MetS and NAFLD.

**Systematic Review Registration:**

https://www.crd.york.ac.uk/prospero/, identifier CRD42021293349

## Introduction

With the development of urbanization and the improvement of living standards, the incidence of diseases related to metabolic disorders is steadily increasing, which makes them serious diseases threatening human health (1–3). Metabolic syndrome (MetS) is defined as a group of complex metabolic disorders characterized by insulin resistance, hypertension, atherogenic dyslipidemia and abdominal obesity, etc. Several societies harmonize that the MetS can be defined when any three or more of the following factors are met: (a) elevated waist circumference based on population-and country-specific definitions; (b) elevated triglycerides or being previously diagnosed as hypertriglyceridemia and taking antihypertriglyceridemia medication; (c) reduced high-density lipoprotein cholesterol (HDL-C) or being previously diagnosed as reduced HDL-C and taking medication for reduced HDL-C; (d) elevated blood pressure (BP) or being previously diagnosed as hypertension and taking antihypertensive medication; (e) raised fasting plasma glucose level or being previously diagnosed as type 2 diabetes and taking antiglycemic medication (4, 5). Non-alcoholic fatty liver disease (NAFLD), a kind of metabolic stress liver injury closely related to insulin resistance and genetic susceptibility, has been regarded as the leading chronic liver disease and primary cause of abnormal liver biochemical indexes found in physical examination (6). The prevalence of NAFLD is increasing worldwide, with an average prevalence of about 24% (7). The close correlation between NAFLD and MetS, and the reciprocal causality between them has been reported. Therefore, some of risk factors and serum diagnostic markers for both NAFLD and MetS may be consistent (8, 9). Current literature has shown there are no drugs available for the treatment of MetS and NAFLD. Owing to significant increased incidence of MetS and NAFLD, it’s essential to seek for new therapeutic agents or targets for those.

Serum bilirubin, mainly originating from the catabolism of hemoglobin in senescent erythrocyte, is commonly used as a biochemical index for the diagnosis of hepatobiliary and metabolic diseases. The conjugation between free bilirubin and UDP-glucuronosyltransferase (UGT) 1A1, which catalyzes the transfer of glucuronic acid, leads to the generation of conjugated bilirubin. For a long time, bilirubin has been deemed as a metabolic waste of iron porphyrin compounds, which means no beneficial effects can be provided by bilirubin. However, latest studies have shown that mildly elevated bilirubin, such as that found in Gilbert’s syndrome(GS), may serve as an important endogenous tissue protector. Meanwhile, it can act as a physiological modulator of oxidative stress and chronic inflammation in MetS (10, 11). In a meta-analysis including 9 observational studies, serum bilirubin levels are demonstrated to be inversely associated with adverse metabolic outcomes. Unfortunately, subgroup analysis was not performed considering that fewer studies were included. In addition, the lack of information about direct bilirubin (DBIL) and indirect bilirubin (IBIL) limits the value of that meta-analysis to evaluate which kind of bilirubin is associated with MetS (12). Both MetS and NAFLD seem to be associated with serum bilirubin, including total bilirubin (TBIL), DBIL, and IBIL. And the increase in bilirubin levels has been demonstrated to be negatively correlated with the prevalence of NAFLD (8, 13–16). Nevertheless, the alternative study based on Mendelian randomization analysis did not find a causal relationship between bilirubin levels and the risk of NAFLD (17, 18). Similarly, Bellarosa et al. reported that bilirubin does not provide protection against MetS and NAFLD in children population with severe obesity (8). What is noteworthy is that the association between bilirubin and NAFLD or MetS in normal-weight adults remains controversial. Importantly, there are no meta-analysis evaluating the association between serum bilirubin and NAFLD currently. In this context, we conducted this meta-analysis to clarify the relationship between serum bilirubin levels and the MetS or NAFLD.

## Materials and Methods

This meta-analysis was designed and implemented according to the Preferred Reporting Items for Systematic Reviews and Meta−Analyses(MOOSE) guidelines [**Supplementary Material 1**], and the search strategy [**Supplementary Material 2**], eligibility criteria and outcomes had been registered in the PROSPERO database (CRD42021293349).

### Search Strategy

Pubmed, Embase and Cochrane Library databases were searched for analyzing the association between serum bilirubin and MetS or NAFLD until November 2021. Subject terms included ‘Non-alcoholic Fatty Liver Disease’, ‘Metabolic Syndrome’, and ‘Bilirubin’, and the random combination of these words were utilized for retrieval. The detailed literature search strategy was shown in supplementary material. Besides, relevant references were also manually searched. The preliminary screening of collected studies was conducted by scanning titles and abstracts. Then, full text was read through to identify the studies that met the inclusion criteria.

### Inclusion Criteria

Studies meeting the following criteria were included (1): randomized controlled trials (RCTs), cross-sectional studies, case-control studies or cohort studies which evaluated the association between TBIL, DBIL or IBIL and MetS or NAFLD (2); similar or identical research protocols were adopted (3); diagnostic criteria of cases were definite (4); comprehensive statistical indicators were provided, such as odds ratio (OR), relative risk (RR), or hazard ratio (HR) with their 95% credible interval (CI).

### Data Extraction and Quality Assessment

Two investigators independently carried out data extraction and quality assessment. Disagreements were reconciled by a third investigator when different opinions exist. Following data were extracted from initial studies: study characteristics (first author, study type, year of publication, country, and follow-up time), patient characteristics (sample size, cases, gender, age, and adjusted covariates), and outcome indicators (OR, RR, HR with their 95% CI). Since the enrolled studies were cross-sectional or cohort studies, Newcastle-Ottawa Quality Assessment Scale (NOS) was used to evaluate the literature quality by two independent reviewers (19). Studies with an NOS score ≥7, 5-6, and <5 were considered as high, fair, and low quality. A score ≥5 indicated adequate quality for inclusion in the present review (12).

### Statistical Analysis

Meta-analysis was implemented using Review Manager 5.3 software (The Cochrane Collaboration, Software Update, Oxford, UK). OR, RR, and HR were defined as the effect indicators, and the point estimates with 95%CI were calculated for these effect indicators. Heterogeneity was assessed using both the chi-square test and I² index, the value of P<0.1 and I²>50% was considered significant. Subsequently, random-effects model was created to incorporate effect indicators when significant heterogeneity existed, otherwise the fixed-effects model would be aqdopted. Subgroup and sensitivity analysis were performed to find out the source of heterogeneity and verify the accuracy of analysis results, respectively. Sensitivity analysis was carried out by excluding studies one by one to identify the studies with significant heterogeneity. Funnel plots was used to assess the possibility of publication bias.

## Results

### Study Selection and Study Characteristics

**Figure 1:**
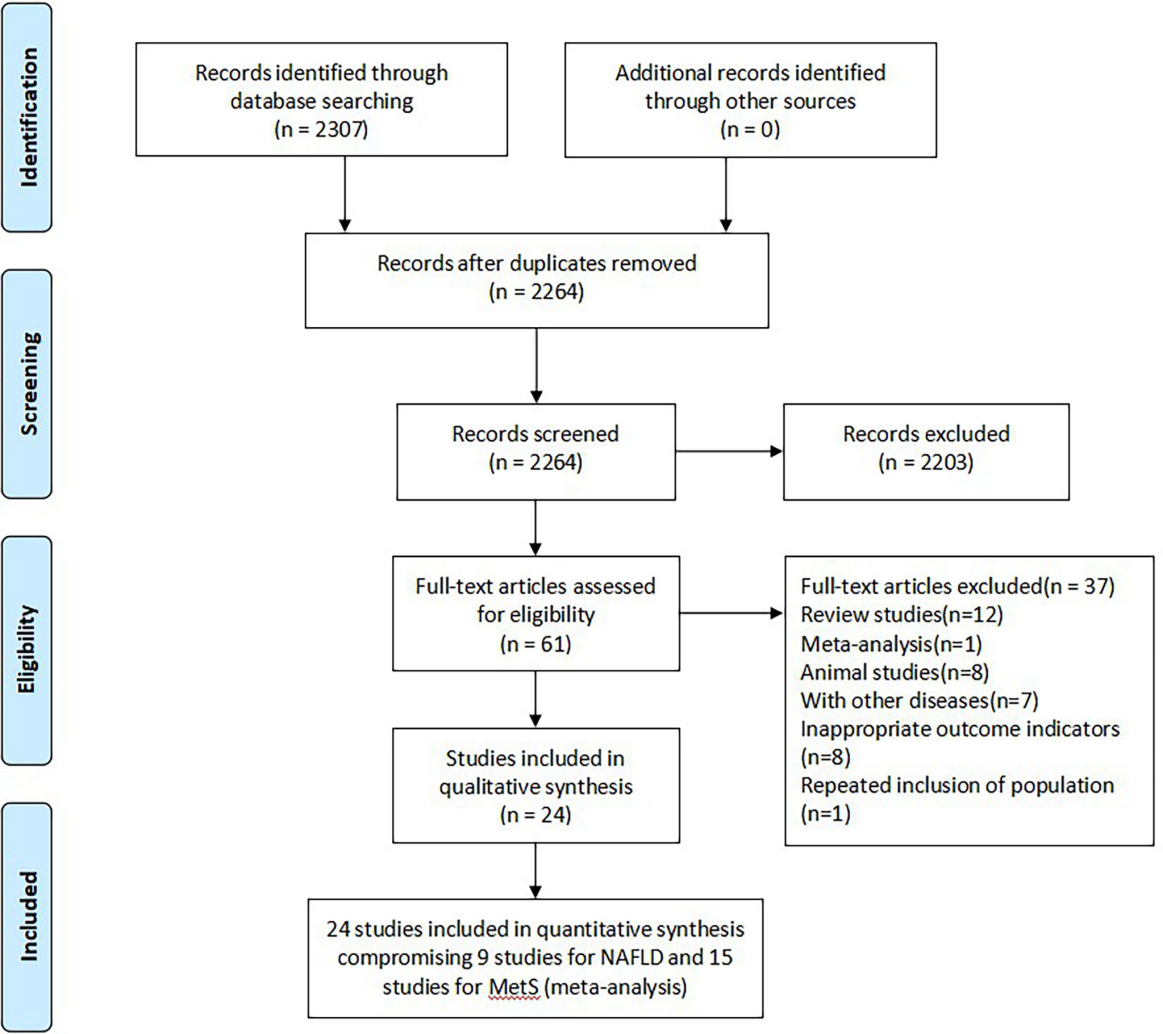
Flow diagram of literature screening and selection. MetS, metabolic syndrome; NAFLD, non-alcoholic fatty liver disease.

A total of 2307 studies were identified based on retrieval strategy, and 43 repetitive articles were excluded by NoteExpress software. After scanning the titles and abstracts, 61 studies were left. Then, we read through the remaining studies in full-text. After that, 101, 517 healthy persons from 24 observational studies including cross-sectional and cohort studies were included in this meta-analysis (13–18, 20–37). The flow diagram of literature screening was shown in **Figure 1**. All included studies were dual-arm studies, of which 15 studies including 11, 696 cases and 6 studies including 9,813 cases evaluated the association between serum bilirubin and MetS (20–34) or NAFLD (13, 14, 17, 18, 35, 36) in health screening population, respectively, while the remaining 3 studies (15, 16, 37) including 997 cases evaluated the association between serum bilirubin and NASH in NAFLD patients. Of note, 15 studies on MetS were conducted in Asian countries included China, Korea and Japan (20–34). Four studies on NAFLD derived from Western countries (15, 16, 18, 35), while the remaining 5 studies derived from Eastern ones (13, 14, 17, 36, 37). The NOS scores of all included literatures were no less than 7 points, indicating high literature quality. The characteristics of included studies were shown in **Table 1**.

**Table 1:** Characteristics of included studies.

Author, year of type/publication (years)	Country	Study follow-up	Year(s) of study	Age(years)	Sample size	Gender (M/F)	Cases	Adjusted covariates	Quality(NOS)
Metabolic syndrome (MetS)									
Hao 2020 (20)	China	Cohort/5.72±1.49	2009-2012	40.3±11.9	565	221/344	204	Age, gender	8
Kawamoto	Japan	Cross-sectional	2014-2017	70±9	893	893/0	451	Age, smoking, alcohol, exercise, presence of CVD,LDL-C, SUA, eGFR, GGT, ALT	
2019 (21)Kawamoto	Japan	Cohort/4	2014-2017	66±9	288	288/0	46		9
2019 (21)									
Li 2017 (22)	China	Cohort/5	2011-2016	45.6±12.7	1339	1339/0	117	Age, alcohol, smoking, exercise,TG, LDL-C	8
Zhong 2017(23)	China	Cross-sectional	2012	73.1±6.6	1728	744/984	484	Age, gender, exercise, smoking, alcohol, TC,ALT	7
Lee 2016 (24)	Korea	Retrospective/>4	2006-2012	50.9	11613	6890/4723	2439	Age, smoking, medication history, ALT, SUA,	8
				(18-89)				eGFR, FPG, diabetes, SBP, WC, BMI	
Chen 2016(25)	China	Cohort/5	2006-2011	45.6±10.0	5258	3262/1996	831	Age, ALT, AST, BUN, WBC, GGT, SUA, gender,	7

### The Association Between Serum Bilirubin and MetS

A total of 15 studies evaluated the association between TBIL, DBIL or IBIL with MetS (20–34). Among them, two studies included both cross-sectional and cohort population (21, 29). To eliminate the influence of gender on analysis results, population were divided into male group (contain only males), female group (contain only females) and gender-neutral group (contain both males and females) for meta-analysis, respectively. When enough studies were included, they were redivided into cross-sectional group and cohort group for subgroup analysis.

### The Association Between TBIL and MetS

**Figure 2:**
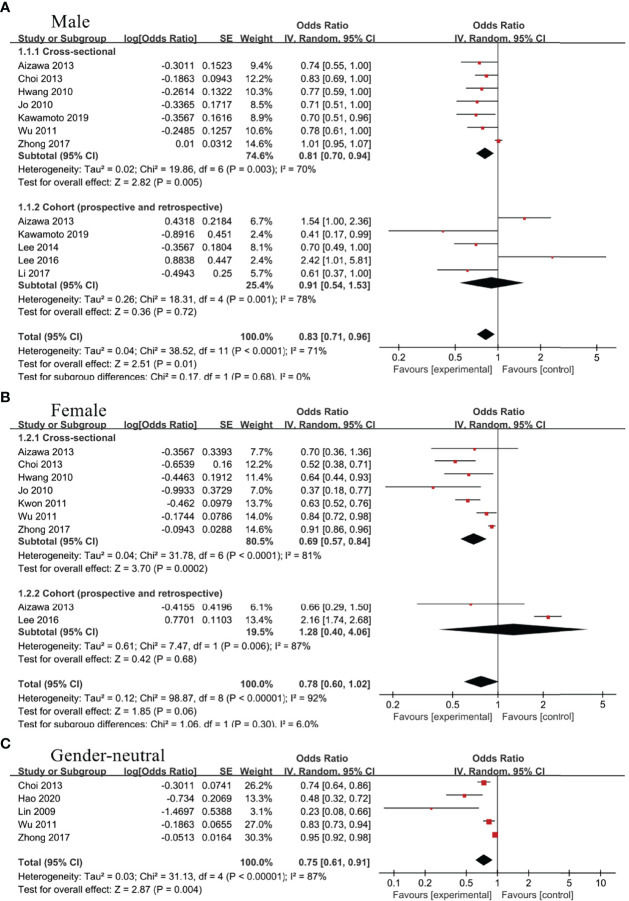
Association of metabolic syndrome (MetS) with total bilirubin (TBIL) among **(A)** male group, **(B)** female group, and **(C)** gender-neutral group.

Among male group, 10 studies evaluated the association between TBIL and MetS, including 7 cross-sectional studies (21, 23, 28–30, 32, 33) and 5 cohort studies (21, 22, 24, 27, 29). There was obvious heterogeneity between included studies, so random effect model was used for analysis. According to our results, TBIL level was inversely associated with MetS in cross-sectional subgroup (OR=0.81, 95%CI=0.70-0.94, P=0.005), whereas no statistical correlation was found in cohort subgroup (OR=0.91, 95%CI=0.54-1.53, P=0.72). Moreover, the pooled results from cross-sectional and cohort studies showed a negative correlation between TBIL and MetS (OR=0.83, 95%CI=0.71-0.96, P=0.01) (**Figure 2A**). Seven cross-sectional studies (23, 28–33) and 2 cohort studies (24, 29) assessed the relationship between TBIL and MetS in female group. Random effect model was adopted for meta-analysis, and the results of cross-sectional subgroup, cohort subgroup and comprehensive analysis displayed negative correlation (OR=0.69, 95%CI=0.57-0.84, P=0.0002), no correlation (OR=1.28, 95%CI=0.40-4.06, P=0.68) and no correlation (OR=0.78, 95%CI=0.60-1.02, P=0.06), respectively (**Figure 2B**) between TBIL and MetS. Random effect model analysis for gender-neutral population showed inverse association between TBIL and MetS (OR=0.75, 95%CI=0.61-0.91, P=0.004) (20, 23, 28, 30, 34) (**Figure 2C**).

### The Association Between DBIL and MetS

**Figure 3:**
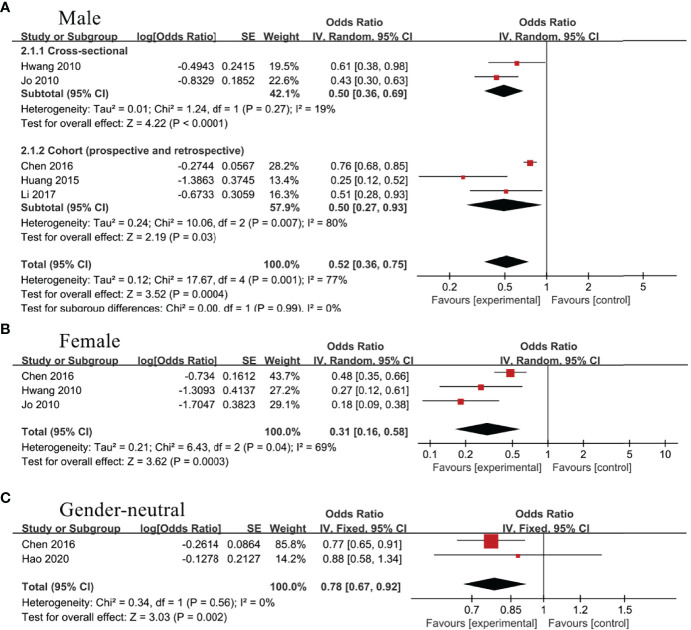
Association of metabolic syndrome (MetS) with direct bilirubin (DBIL) among **(A)** male group, **(B)** female group, and **(C)** gender-neutral group.

Two cross-sectional studies (32, 33) and 3 cohort studies (22, 25, 26) evaluated the association between DBIL and MetS in male group. Random effect model was adopted and meta-analysis showed that DBIL were negative correlated with MetS in both cross-sectional (OR=0.50, 95%CI=0.36-0.69, P<0.0001) and cohort subgroup (OR=0.50, 95%CI=0.27-0.93, P=0.03) (**Figure 3A**). Additionally, the inverse association between DBIL and MetS was found in male (OR=0.52, 95%CI=0.36-0.75, P=0.0004) (22, 25, 26, 32, 33), female (OR=0.31, 95%CI=0.16-0.58, P=0.0003) (25, 32, 33)(**Figure 3B**) and gender-neutral population (OR=0.78, 95%CI=0.67-0.92, P=0.002) (20, 25) (**Figure 3C**), regardless of the study type.

### The Association Between IBIL and Met

**Figure 4:**
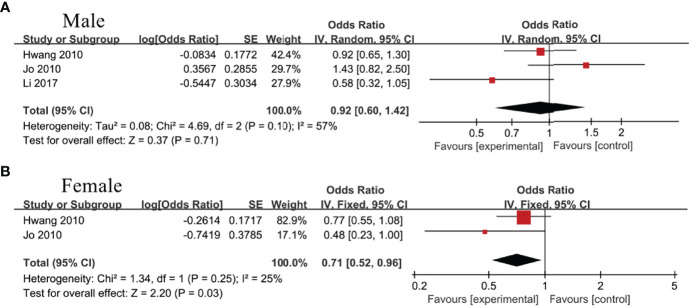
Association of metabolic syndrome (MetS) with indirect bilirubin (IBIL) among **(A)** male group and **(B)** female group.

Three studies (22, 32, 33) including male population only and 2 studies (32, 33) including female population only analyzed the association between IBIL and MetS. As a result, IBIL level was inversely associated with MetS in female group (OR=0.71, 95%CI=0.52-0.96, P=0.03) (**Figure 4B**), whereas no statistical correlation was noticed in male group (OR=0.92, 95%CI=0.60-1.42, P=0.71) (**Figure 4A**).

### The Association Between Serum Bilirubin and NAFLD

Nine studies evaluated the association between TBIL, DBIL or IBIL and NAFLD (13–18, 35–37). In view of the inconsistence in recruited populations and enough sample size, study population was subdivided into gender-neutral and male divisions for TBIL and DBIL sub-groups. And the random effect model was utilized for separate meta-analysis. There was no obvious heterogeneity in IBIL subgroup, so fixed effect model was adopted.

### The Association Between TBIL and NAFLD

**Figure 5:**
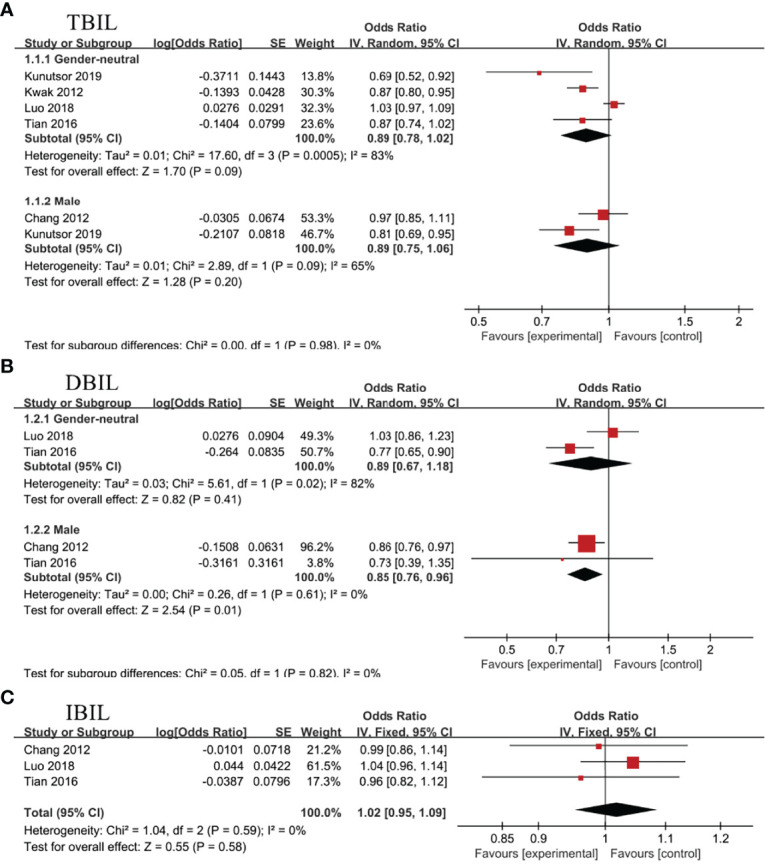
Association of non-alcoholic fatty liver disease (NAFLD) with **(A)** total bilirubin (TBIL), **(B)** direct bilirubin (DBIL), and **(C)** indirect bilirubin (IBIL).

Four studies (13, 14, 17, 18)and 2 studies (18, 36), respectively, evaluated the association between TBIL and NAFLD in gender-neutral and male subgroup. Random effect model analysis showed no statistical correlation between TBIL and NAFLD, regardless of the subgroups, OR=0.89, 95%CI=0.78-1.02, P=0.09 for gender-neutral subgroup; OR=0.89, 95%CI=0.75-1.06, P=0.20 for male subgroup (**Figure 5A**).

### The Association Between DBIL and NAFLD

In gender-neutral subgroup, 2 studies analyzed the connection between DBIL and NAFLD (14, 17), showing no statistical association (OR=0.89, 95%CI=0.67-1.18, P=0.41). Nevertheless, negative correlation between DBIL and NAFLD (OR=0.85, 95%CI=0.76-0.96, P=0.01) was noticed in male subgroup (14, 36)(**Figure 5B**).

### The Association Between IBIL and NAFLD

**Figure 6:**
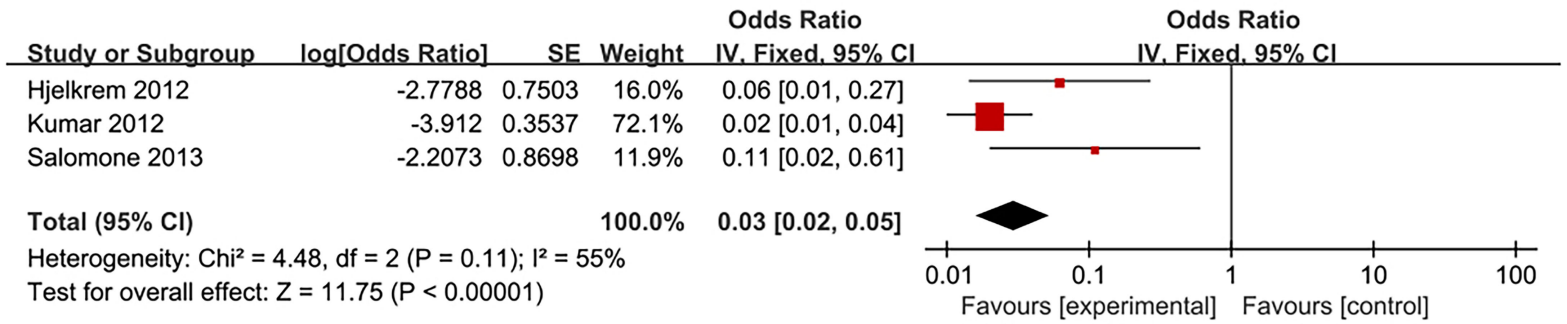
Association of non-alcoholic steatohepatitis (NASH) with indirect bilirubin (IBIL).

Three studies evaluated the association between IBIL and NAFLD in health screening population (14, 17, 36). Meta-analysis revealed no statistical association between IBIL and the incidence rate of NAFLD (OR=1.02, 95%CI=0.95-1.09, P=0.58) (**Figure 5C**). Subsequently, further analysis was conducted to explore the correlation between IBIL and non-alcoholic steatohepatitis (NASH) in NAFLD patients. Random effect model analysis including 3 studies (15, 16, 37) manifested inverse association between IBIL and NASH (OR=0.03, 95%CI=0.02-0.05, P<0.00001) (**Figure 6**).

### Sensitivity Analysis

To estimate the influence of single study on overall results of meta-analysis, sensitivity analysis was carried out by excluding studies one by one. In association analysis between TBIL and NAFLD, we temporarily excluded Luo’s study (P=0.30, I²=17%) and re-analyzed remaining studies. Fixed effect model analysis based on remaining studies showed negative correlation between TBIL and NAFLD in gender-neutral subgroup (OR=0.86, 95%CI=0.80-0.92, P<0.0001), which was different than previous analysis. Additionally, sensitibity analysis based on other outcome indicators all showed no significant changes after deleting each trial, which confirmed the rationality and reliability of our meta-analysis.

### Publication Bias

**Figure 7:**
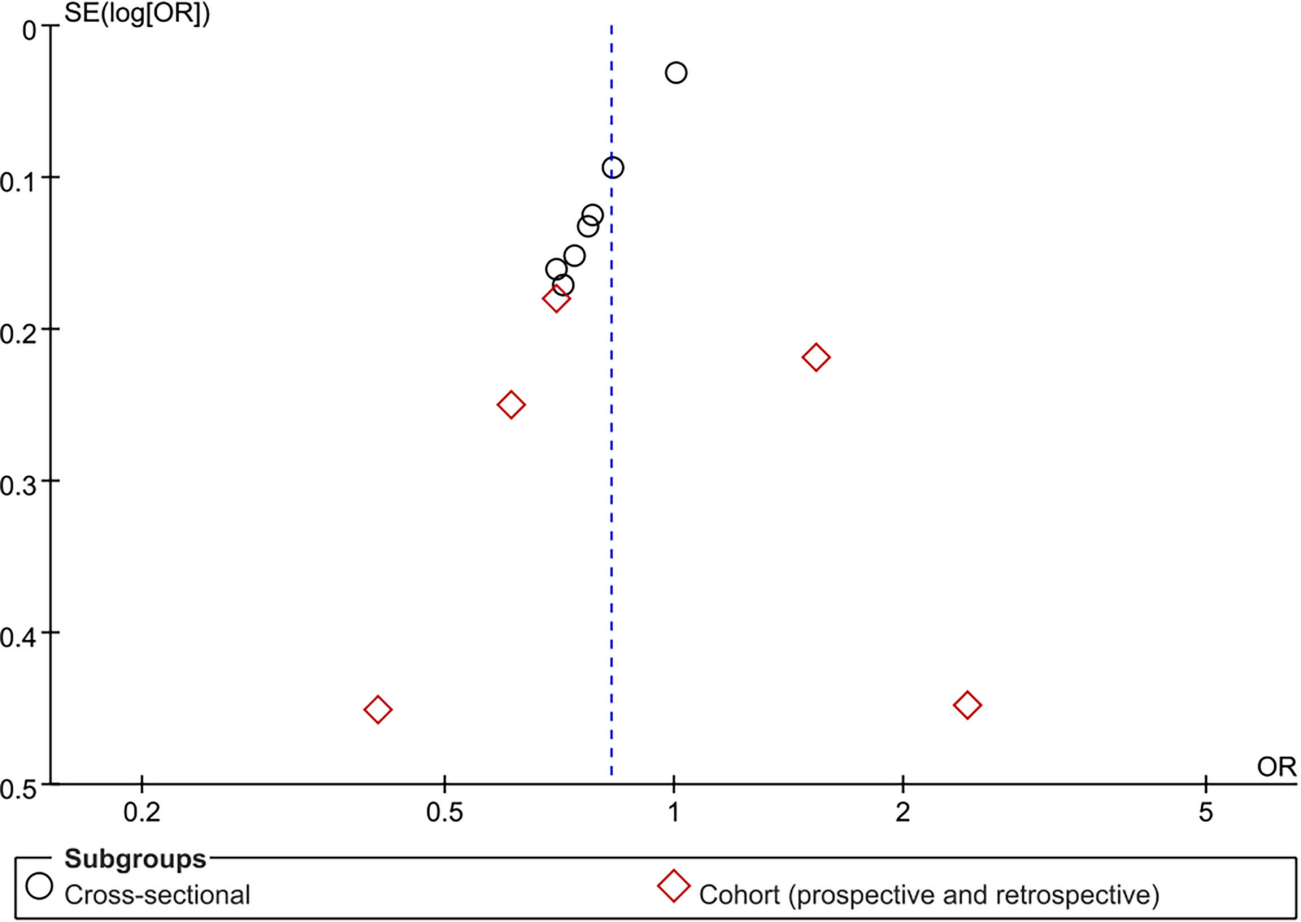
Funnel plot for association of metabolic syndrome (MetS) with total bilirubin (TBIL) among male group.

Publication bias analysis based on the association between TBIL and MetS in male group is more convincing and accurate. Funnel plot was drawn for MetS with TBIL in male group as an outcome indicator, and it was found that the left and right distributions of each study site were asymmetrical, suggesting the possible existence of publication bias (**Figure 7**). The other results of publication bias analysis are shown in **Supplementary Material 3**.

## Discussion

MetS is associated with an increased risk of cardiovascular disease and all-cause mortality (1). NAFLD, characterized by fat degeneration and accumulation in hepatocyte, is regarded as the “liver manifestation of metabolic syndrome”. Owing to significant increased incidence of MetS and NAFLD (1, 38), it’s essential to seek for new therapeutic agents or targets for MetS and NAFLD. At present, hyperbilirubinemia is considered to play a beneficial protective role in numerous oxidative stress and inflammation-related diseases, such as coronary heart disease, diabetes, and stroke (11, 39, 40). What relations have been existed between bilirubin and MetS or NAFLD, and whether hyperbilirubinemia could lower the risk of MetS or NAFLD, however, needs further discussion.

Except for MetS, this study is the first to systematically review and summarize published studies in order to assess the correlation between bilirubin subtypes and NAFLD through meta-analysis. Serum TBIL is inversely associated with MetS in male and gender-neutral group, but not in female. However, the inconsistency existed for the analysis results derived from the cross-sectional and cohort study when we evaluated the association between TBIL and MetS in male or female group There were 7 cross-sectional studies in both male and female group, while 5 cohort studies were included in male and 2 cohort studies in female, respectively. Thus, we speculated that the reason for the inconsistency may be ascribed to the differences in the number of cross-sectional and cohort studies. Fewer cohort studies might bring about the inconsistency. DBIL exhibits an inverse association with MetS, regardless of gender. IBIL displays a negative correlation with MetS in females but not in males. No stastistical correlation is found between TBIL and NAFLD. DBIL is negatively correlated with NAFLD in male subgroup. IBIL manifests an inverse association with NASH in NAFLD patients.

Bilirubin and its precursor biliverdin can increase the antioxidant activity of vascular endothelial cells (41). Bilirubin can also increase insulin sensitivity by regulating levels of cholesterol metabolism, adipokines and peroxisome proliferator-activated receptor γ (PPARγ) (39). What’s more, it can selectively bind to proliferator-activated receptor α (PPARα), causing the decrease in lipid accumulation by increasing the number and function of mitochondria (40, 42, 43). In this analyses, an inverse association was found between TBIL levels and MetS among male and gender-neutral group. In females, TBIL tends to be negatively associated with MetS, although no statistical correlation is found between them(P=0.06). In addition, an inverse association between TBIL and MetS exists in cross-sectional subgroup, but not in cohort subgroup. Insufficient number of included studies in cohort subgroup could affect the accuracy of overall results. Therefore, in line with previous reports, we confirm a protective role of TBIL in MetS (12).

An obvious negative correlation between DBIL and MetS was revealed in all subgroups, regardless of gender and study type. Moreover, correlation between DBIL and MetS is closer than that between IBIL and MetS. As we know, DBIL tends to build looser bound with albumin than IBIL. Hence, it is easier for DBIL to separate from albumin than IBIL. As a result, DBIL might directly act on target organs and molecules (44). Collectively, DBIL may possess better prognostic value than IBIL according to the analysis conducted by we and other investigators (32, 33).

In general, average bilirubin levels in males are slightly higher than those in females. GS, an inherited metabolic liver disease characterized by IBIL elevation, is also most frequently occurs in young males. This discrepancy may be explained by a gap in life style and the effect of sex hormines on the glucuronic acid (45, 46). Hwang et al. demonstrated that all 3 types bilirubin levels are inversely relevant with MetS in females, while DBIL exhibits significant inverse association with MetS in males, after adjusting for the confounding variables including lifestyle (32). Another investigation indicated that all bilirubin subtypes are negatively correlated with MetS in Korean men and women. Nevertheless, this significant inverse correlation between TBIL or IBIL and MetS vanishes according to the adjusted multivariate analysis model (adjusting for age, smoking status, alcohol consumption and so on) (33). In this analysis, we adjusted for the potential confounding factors such as age, gender, life styles, etc. Our data showed that all the studies type of DBIL were inversely related with the MetS in male whereas the IBIL was only inversely related with the MetS in female. These results likely indicate that DBIL is more related to the MetS than the other subtypes of bilirubin in male, and the protective effect of IBIL against MetS is more pronounced in female group. This is partially consistent with Hwang’s findings (32). Apart from uridine diphosphate-glucuronosyltransferase (UGT1A1), the protective effect of bilirubin may be also related to other metabolic enzymes that can regulate the bilirubin metabolism, such as heme oxygenase (HO). Bilirubin is produced under the action of HO, the rate-limiting enzyme of heme catabolism. Downregulation of HO activity inhibits bilirubin production (47). Notably, the gender difference exists in HO activities, which is related to oxidative stress (48) or high iron storage (49). This finding can be used, at least in part, to explain the reasons for the different effects of bilirubin on men and women. However, the exact mechanism is still needed to be explored. Besides, limited number of included studies may reduce the analysis accuracy.

NAFLD does not only refer to the fat accumulation in the liver caused by excessive free fatty acids, but also involves numerous metabolic problems such as oxidative stress, insulin resistance, and mitochondrial dysfunction. Furthermore, a wide spectrum of histological lesions ranging from pure hepatic steatosis to NASH are included in NAFLD. Various laboratory and clinical studies have demonstrated that bilirubin most likely reduces hepatic lipid accumulation by increasing PPARα activation and inhibiting PPARγ transcriptional activity in humanized mice with the Gilbert’s polymorphism (HuUGT*28) or humans with GS (39, 40, 50, 51). According to our analysis, the negative correlation between DBIL and NAFLD only exists in male subgroup, while no significant associations between all bilirubin subtypes with NAFLD is found in gender-neutral group. Furthermore, DBIL rather than other bilirubin subtypes manifests significiant inverse association with MetS or NAFLD in male population. Although no significant relation between IBIL and NAFLD is noticed in health screening population, an inverse association is reported between IBIL and NASH occurring in NAFLD patientsis. These findings are not compatible with the results provided by Luo et al. We think this inconsistence can be ascribed to the following factors. In Luo’s study, NAFLD was diagnosed based on liver ultrasonography rather than liver biopsy which is regarded as,the gold standard for NAFLD diagnosis (52, 53). As a result, mild fatty liver might be missed considering the insensitiveness originating from ultrasonography. In addition, Luo’s study mainly enrolled mid-aged adults with lower incidence of NAFLD, therefore bringing about the risk to draw an biased conclusion (17). In this context, three separate investigation diagnosed NAFLD based on liver biopsy results, and the authors found that IBIL is inversely associated with the severity of liver damage in NASH patients (15, 16, 37). Oxidative stress has been documented to promote the progression from hepatic steatosis to NASH (38, 53). In view of the potent oxidation resistance, it is conceivable that IBIL may provide a protective effect through antioxidant activity in lipotoxic diseases such as NAFLD. Even through, the association between serum IBIL and NAFLD and the underlying mechanism behind this association still need to be explored. Nowadays, studies on association between TBIL or DBIL and NASH in NAFLD patients is insufficient.

Women at reproductive period have a different metabolic status from those at post-menopause. It may reflect the effects of decreased estrogen levels, which have a certain impact on lipid metabolism and insulin resistance. In addition, estrogen deficiency hastens the development of hepatic steatosis and the progression of hepatic fibrosis (53, 54). Serum bilirubin levels in post-menopause may be higher than pre-menopause, which is also related to estrogens deficiency (45, 46). Elevated bilirubin levels have been reported to be closely related to decreased prevalence rate of MetS or NAFLD (12, 13). And our results partially support this finding. Nevertheless, elevated bilirubin levels may not counteract the effect of estrogen deficiency in postmenopausal women. Thus, the prevalence rates of MetS and NAFLD are significantly higher in postmenopausal women than pre-menopausal women (1, 2, 7). For premenopausal women, not only bilirubin but also estrogen can protect from developing MetS and NAFLD. Because of the lack of detailed information for determining whether a female participant is in post-menopause or not, it is unfeasible to conduct subgroup analysis based on this factor. Further research is needed to be carried out to clarify this issue.

Why the association is inconsistent between serum bilirubin and MetS or NAFLD? The possible reasons are as follows: Primarily, except for the liver, other factors such as other tissues, gene, et. are also involved important role in the pathogenesis and development of MetS or NAFLD (55). For instance, steatosis in PNPLA3-associated NAFLD is not accompanied by features of MetS, while PNPLA3-uncorrelated NAFLD closely resembles MetS with regards to its causes and consequences (56). There is a basic research showed that bilirubin deficiency renders mice susceptible to hepatic steatosis in the absence of insulin resistance. It adopted a kind of contrarian strategy to prove that the pathogenesis of MetS and NAFLD is not exactly identically (57). In addition, the variety of definitions of MetS and the variety of diagnostic methods for NAFLD in different studies is also make it challenging that assess the consistent-association of serum bilirubin with MetS and NAFLD.

The inverse correlation between serum bilirubin and MetS or NAFLD suggests that bilirubin might be utilized as a potential and promising strategy to assist in lowering the risk of developing MetS and NAFLD. Exogenous bilirubin supplement is the most direct way to prevent the occurrence of MetS and NAFLD. So far, several studies have attempted to carry out targeted therapy for cancer, inflammation and vascular diseases utilizing bilirubin nanoparticles (BRNP) or bilirubin coated stents (58). And the preliminary efficacy is promising. Moreover, basic research shows that BRNP reduces diet-induced hepatic steatosis (59). On the other hand, increasing endogenous bilirubin production is also a feasible treatment strategy. In this regard, inducing “iatrogenic Gilbert syndrome” by uricosuric drug-probenecid has been demonstrated to be capable of reducing the liver gluconaldehyde acidification activity followed by the increase in serum bilirubin (60). Curcumin supplemented by diet has been documented to increase bilirubin levels through targeting HO-1. These strategies have been proven to be effective and safe *in vitro* and *in vivo*. Therefore, increasing bilirubin levels may be an advantageous treatment strategy for MetS and NAFLD (47).

Although the stratification has been executed as far as possible, the shortcomings exist in this meta-analysis. Firstly, the absence of RCTs or prospective cohort studies brings down the credibility of analysis results. Secondly, the classification criteria for serum bilirubin are not identical among different studies, which may partially account for the existence of heterogeneity. Thirdly, insufficient studies on the association between TBIL or DBIL and NASH in NAFLD patients that impairs the credibility and clinical value of this analysis. Fourthly, the studies on MetS were all conducted in Asian countries included China, Korea and Japan. There are no obvious difference in geographical location, dietary patterns, figures, and life styles among these coutryies. Thus, the dietary patterns may have little impact on the results related to MetS in this meta analysis. In terms of NAFLD, four studies derived from Western countries and five studies derived from Eastern countries. Nevertheless, it is unfeasible to conduct subgroup analysis in view of lacking detailed information on diet. Fifthly, in terms of age, most studies included in our meta analysis refer to populations at all ages. However, most original studies had adjusted age as a covariate. For the remaining studies, the lack of detailed information on age makes it infeasible to carry out subgroup analysis on age. Meanwhile, lacking of detailed information on menopause, it is infeasible to conduct subgroup analysis on pre or post-menopause. Finally, follow-up time for each included study is inconsistent or unclear, therefore underscoring the incidence of MetS or NAFLD. Consequently, more high-quality, large-scale, prospective and long-term follow-up studies are urgently needed.

## Conclusion

In brief, our meta-analysis indicates that serum TBIL and DBIL levels, especially serum DBIL levels, supporting an inverse connection with MetS, Moreover, serum IBIL could decrease the onset of NASH in NAFLD patients. Therefore, appropriately elevated serum bilirubin levels seem to reduce the risk of MetS and NAFLD. Regulation of bilirubin metabolic pathways may be a potential strategy and exogenous bilirubin supplement may be a medicine to assist in lowering the risk of developing MetS and NAFLD. Bilirubin is still far from being used in the clinic at present. Large-scale prospective and high-quality animal or clinical studies are required to establish to investigate the association and potential prevention of bilirubin on MetS or NAFLD.

## Data Availability Statement

The original contributions presented in the study are included in the article/**Supplementary Material**. Further inquiries can be directed to the corresponding author.

## Author Contribution

CL and ZY wrote and amended the original draft. LB and WH participated in extracting and analyzing the data. ST and WZ searched literature and produced the tables, figures. XC, ZH and ZD gave critical revisions, and final approval of the article. SZ contributed to design the study, interpretation of data, and the final approval of the article. All authors contributed to the article and approved the submitted version.

## Funding

The current study was supported by a research grant from Beijing Municipal Administration of Hospitals Clinical medicine Development of Special Funding Support (ZYLX202125), Capital Health development Scientific Research project (2022–1–2182), Natural Science Foundation of Beijing Municipality (7202068), Beijing Advanced Innovation Center for Big Data-Based Precision Medicine (1212040205).

## Conflict of Interest

The authors declare that the research was conducted in the absence of any commercial or financial relationships that could be construed as a potential conflict of interest.

## Publisher’s Note

All claims expressed in this article are solely those of the authors and do not necessarily represent those of their affiliated organizations, or those of the publisher, the editors and the reviewers. Any product that may be evaluated in this article, or claim that may be made by its manufacturer, is not guaranteed or endorsed by the publisher.

## References

[1] Hirode G Wong RJ . Trends in the Prevalence of Metabolic Syndrome in the United States, 2011-2016. JAMA (2020) 323(24):2526–8. doi: 10.1001/jama.2020.4501 PMC7312413 32573660

[2] Aguilar M Bhuket T Torres S Liu B Wong RJ . Prevalence of the Metabolic Syndrome in the United States, 2003-2012. JAMA (2015) 313(19):1973–4. doi: 10.1001/jama.2015.4260 25988468

[3] Bishehsari F Voigt RM Keshavarzian A . Circadian Rhythms and the Gut Microbiota: From the Metabolic Syndrome to Cancer. Nat Rev Endocrinol (2020) 16(12):731–9. doi: 10.1038/s41574-020-00427-4 PMC8085809 33106657

[4] Emir M Dimitri PM Christos M . Non-Alcoholic Fatty Liver Disease, Insulin Resistance, Metabolic Syndrome and Their Association With Vascular Risk. Metabolism: Clin Exp (2021) 06(119):154770. doi: 10.1016/j.metabol.2021.154770 33864798

[5] Peter MN Jaakko T Lars R . The Metabolic Syndrome - What Is It and How Should It Be Managed? Eur J Prev Cardiol (2019) 26(2_suppl):33–46. doi: 10.1177/2047487319886404 31766917

[6] Svegliati-Baroni G Pierantonelli I Torquato P Marinelli R Ferreri C Chatgilialoglu C . Lipidomic Biomarkers and Mechanisms of Lipotoxicity in Non-Alcoholic Fatty Liver Disease. Free Radical Biol Med (2019) 144:293–309. doi: 10.1016/j.freeradbiomed.2019.05.029 31152791

[7] Younossi ZM Koenig AB Abdelatif D Fazel Y Henry L Wymer M . Global Epidemiology of Nonalcoholic Fatty Liver Disease-Meta-Analytic Assessment of Prevalence, Incidence, and Outcomes. Hepatology (2016) 64(1):73–84. doi: 10.1002/hep.28431 26707365

[8] Bellarosa C Bedogni G Bianco A Cicolini S Caroli D Tiribelli C . Association of Serum Bilirubin Level With Metabolic Syndrome and Non-Alcoholic Fatty Liver Disease: A Cross-Sectional Study of 1672 Obese Children. J Clin Med (2021) 10(13):1–13. doi: 10.3390/jcm10132812 PMC8268762 34202304

[9] Sookoian S Pirola CJ . Review Article: Shared Disease Mechanisms Between Non-Alcoholic Fatty Liver Disease and Metabolic Syndrome - Translating Knowledge From Systems Biology to the Bedside. Alimentary Pharmacol Ther (2019) 49(5):516–27. doi: 10.1111/apt.15163 30714632

[10] Vitek L . Bilirubin as a Signaling Molecule. Med Res Rev (2020) 40(4):1335–51. doi: 10.1002/med.21660 32017160

[11] Fujiwara R Haag M Schaeffeler E Nies AT Zanger UM Schwab M . Systemic Regulation of Bilirubin Homeostasis: Potential Benefits of Hyperbilirubinemia. Hepatology (2018) 67(4):1609–19. doi: 10.1002/hep.29599 29059457

[12] Nano J Muka T Cepeda M Voortman T Dhana K Brahimaj A . Association of Circulating Total Bilirubin With the Metabolic Syndrome and Type 2 Diabetes: A Systematic Review and Meta-Analysis of Observational Evidence. Diabetes Metab (2016) 42(6):389–97. doi: 10.1016/j.diabet.2016.06.002 27396752

[13] Kwak MS Kim D Chung GE Kang SJ Park MJ Kim YJ . Serum Bilirubin Levels Are Inversely Associated With Nonalcoholic Fatty Liver Disease. Clin Mol Hepatol (2012) 18(4):383–90. doi: 10.3350/cmh.2012.18.4.383 PMC3540375 23323254

[14] Tian J Zhong R Liu C Tang Y Gong J Chang J . Zhang Y Et Al: Association Between Bilirubin and Risk of Non-Alcoholic Fatty Liver Disease Based on a Prospective Cohort Study. Sci Rep (2016) 6:31006. doi: 10.1038/srep31006 27484402 PMC4975069

[15] Salomone F Li Volti G Rosso C Grosso G Bugianesi E . Unconjugated Bilirubin, a Potent Endogenous Antioxidant, Is Decreased in Patients With Non-Alcoholic Steatohepatitis and Advanced Fibrosis. J Gastroenterol Hepatol (2013) 28(7):1202–8. doi: 10.1111/jgh.12155 23425054

[16] Hjelkrem M Morales A Williams CD Harrison SA . Unconjugated Hyperbilirubinemia Is Inversely Associated With Non-Alcoholic Steatohepatitis (NASH). Alimentary Pharmacol Ther (2012) 35(12):1416–23. doi: 10.1111/j.1365-2036.2012.05114.x 22540836

[17] Luo L An P Jia X Yue X Zheng S Liu S . Genetically Regulated Bilirubin and Risk of Non-Alcoholic Fatty Liver Disease: A Mendelian Randomization Study. Front Genet (2018) 9:662. doi: 10.3389/fgene.2018.00662 30619479 PMC6305545

[18] Kunutsor SK Frysz M Verweij N Kieneker LM Bakker SJL Dullaart RPF . Circulating Total Bilirubin and Risk of Non-Alcoholic Fatty Liver Disease in the PREVEND Study: Observational Findings and a Mendelian Randomization Study. Eur J Epidemiol (2020) 35(2):123–37. doi: 10.1007/s10654-019-00589-0 PMC7125247 31773475

[19] Stang A . Critical Evaluation of the Newcastle-Ottawa Scale for the Assessment of the Quality of Nonrandomized Studies in Meta-Analyses. Eur J Epidemiol (2010) 25(9):603–5. doi: 10.1007/s10654-010-9491-z 20652370

[20] Hao H Guo H Ma RL Yan YZ Hu YH Ma JL . Association of Total Bilirubin and Indirect Bilirubin Content With Metabolic Syndrome Among Kazakhs in Xinjiang. BMC Endocr Disord (2020) 20(1):110. doi: 10.1186/s12902-020-00563-y 32698889 PMC7376964

[21] Kawamoto R Kikuchi A Akase T Ninomiya D Kasai Y Ohtsuka N . Total Bilirubin Independently Predicts Incident Metabolic Syndrome Among Community-Dwelling Women. Diabetes Metab Syndr (2019) 13(2):1329–34. doi: 10.1016/j.dsx.2019.02.009 31336487

[22] Li XH Lin HY Guan LY Peng H Wen MM Cao YQ . Direct Bilirubin Levels and Risk of Metabolic Syndrome in Healthy Chinese Men. BioMed Res Int (2017) 2017:9621615. doi: 10.1155/2017/9621615 29423413 PMC5750483

[23] Zhong P Sun DM Wu DH Li TM Liu XY Liu HY . Serum Total Bilirubin Levels Are Negatively Correlated With Metabolic Syndrome in Aged Chinese Women: A Community-Based Study. Braz J Med Biol Res (2017) 50(2):e5252. doi: 10.1590/1414-431X20165252 28146216 PMC5304216

[24] Lee YB Lee SE Jun JE Jee JH Bae JC Jin SM . Change in Serum Bilirubin Level as a Predictor of Incident Metabolic Syndrome. PLoS One (2016) 11(12):e0168253. doi: 10.1371/journal.pone.0168253 27936224 PMC5148095

[25] Chen Q Xiao J Zhang P Chen L Chen X Wang S . [Association Between Serum Direct Bilirubin With Metabolic Syndrome and Its Components Based on a Longitudinal Health Check-Up Study]. Zhonghua Liu Xing Bing Xue Za Zhi (2016) 37(4):486–90. doi: 10.3760/cma.j.issn.0254-6450.2016.04.009 27087211

[26] Huang SS Chan WL Leu HB Huang PH Lin SJ Chen JW . Serum Bilirubin Levels Predict Future Development of Metabolic Syndrome in Healthy Middle-Aged Nonsmoking Men. Am J Med (2015) 128(10):1138.e1135–1141. doi: 10.1016/j.amjmed.2015.04.019 25912203

[27] Lee MJ Jung CH Kang YM Hwang JY Jang JE Leem J . Serum Bilirubin as a Predictor of Incident Metabolic Syndrome: A 4-Year Retrospective Longitudinal Study of 6205 Initially Healthy Korean Men. Diabetes Metab (2014) 40(4):305–9. doi: 10.1016/j.diabet.2014.04.006 24951082

[28] Choi SH Yun KE Choi HJ . Relationships Between Serum Total Bilirubin Levels and Metabolic Syndrome in Korean Adults. Nutr Metab Cardiovasc Dis (2013) 23(1):31–7. doi: 10.1016/j.numecd.2011.03.001 21703835

[29] Oda E Aizawa Y . Total Bilirubin Is Inversely Associated With Metabolic Syndrome but Not a Risk Factor for Metabolic Syndrome in Japanese Men and Women. Acta Diabetol (2013) 50(3):417–22. doi: 10.1007/s00592-012-0447-5 23224110

[30] Wu Y Li M Xu M Bi Y Li X Chen Y . Low Serum Total Bilirubin Concentrations Are Associated With Increased Prevalence of Metabolic Syndrome in Chinese. J Diabetes (2011) 3(3):217–24. doi: 10.1111/j.1753-0407.2011.00138.x 21631904

[31] Kwon KM Kam JH Kim MY Kim MY Chung CH Kim JK . Inverse Association Between Total Bilirubin and Metabolic Syndrome in Rural Korean Women. J Womens Health (Larchmt) (2011) 20(6):963–9. doi: 10.1089/jwh.2010.2453 21671781

[32] Hwang HJ Kim SH . Inverse Relationship Between Fasting Direct Bilirubin and Metabolic Syndrome in Korean Adults. Clin Chim Acta (2010) 411(19-20):1496–501. doi: 10.1016/j.cca.2010.06.003 20542021

[33] Jo J Yun JE Lee H Kimm H Jee SH . Total, Direct, and Indirect Serum Bilirubin Concentrations and Metabolic Syndrome Among the Korean Population. Endocrine (2010) 39(2):182–9. doi: 10.1007/s12020-010-9417-2 21116740

[34] Lin LY Kuo HK Hwang JJ Lai LP Chiang FT Tseng CD . Serum Bilirubin Is Inversely Associated With Insulin Resistance and Metabolic Syndrome Among Children and Adolescents. Atherosclerosis (2009) 203(2):563–8. doi: 10.1016/j.atherosclerosis.2008.07.021 18775539

[35] Puri K Nobili V Melville K Corte CD Sartorelli MR Lopez R . Serum Bilirubin Level Is Inversely Associated With Nonalcoholic Steatohepatitis in Children. J Pediatr Gastroenterol Nutr (2013) 57(1):114–8. doi: 10.1097/MPG.0b013e318291fefe 23518490

[36] Chang Y Ryu S Zhang Y Son HJ Kim JY Cho J . A Cohort Study of Serum Bilirubin Levels and Incident Non-Alcoholic Fatty Liver Disease in Middle Aged Korean Workers. PLoS One (2012) 7(5):e37241. doi: 10.1371/journal.pone.0037241 22615952 PMC3352875

[37] Kumar R Rastogi A Maras JS Sarin SK . Unconjugated Hyperbilirubinemia in Patients With Non-Alcoholic Fatty Liver Disease: A Favorable Endogenous Tesponse. Clin Biochem (2012) 45(3):272–4. doi: 10.1016/j.clinbiochem.2011.11.017 22198578

[38] Lazarus JV Colombo M Cortez-Pinto H Huang TT Miller V Ninburg M . NAFLD - Sounding the Alarm on a Silent Epidemic. Nat Rev Gastroenterol Hepatol (2020) 17(7):377–9. doi: 10.1038/s41575-020-0315-7 32514153

[39] Liu J Dong H Zhang Y Cao M Song L Pan Q . Bilirubin Increases Insulin Sensitivity by Regulating Cholesterol Metabolism, Adipokines and PPARgamma Levels. Sci Rep (2015) 5:9886. doi: 10.1038/srep09886 26017184 PMC4446899

[40] Molzer C Wallner M Kern C Tosevska A Schwarz U Zadnikar R . Features of an Altered AMPK Metabolic Pathway in Gilbert’s Syndrome, and Its Role in Metabolic Health. Sci Rep (2016) 6:30051. doi: 10.1038/srep30051 27444220 PMC4956769

[41] Ziberna L Martelanc M Franko M Passamonti S . Bilirubin Is an Endogenous Antioxidant in Human Vascular Endothelial Cells. Sci Rep (2016) 6:29240. doi: 10.1038/srep29240 27381978 PMC4933905

[42] Gordon DM Adeosun SO Ngwudike SI Anderson CD Hall JE Hinds TD Jr. . CRISPR Cas9-Mediated Deletion of Biliverdin Reductase a (BVRA) in Mouse Liver Cells Induces Oxidative Stress and Lipid Accumulation. Arch Biochem Biophys (2019) 672:108072. doi: 10.1016/j.abb.2019.108072 31422074 PMC6718297

[43] Montaigne D Butruille L Staels B . PPAR Control of Metabolism and Cardiovascular Functions. Nat Rev Cardiol (2021) 18(12):809–23. doi: 10.1038/s41569-021-00569-6 34127848

[44] Tatsuyoshi Nakagami KT . Taroh Kinoshita B, Seiji Morisawa: A Beneficial Role of Bile Pigments as an Endogenous Tissue Protector: Anticomplement Effects of Biliverdin and Conjugated Bilirubin. Biochim Biophys Acta (1993) 1158(2):189–93. doi: 10.1016/0304-4165(93)90013-x 8399320

[45] Gentile S Tiribelli C Baldini G Lunazzi G Sottocasa GL . Sex Differences of Nicotinate-Induced Hyperbilirubinemia in Gilbert’s Syndrome. Implication of Bilitranslocase Function. J Hepatol (1985) 1(4):417–29. doi: 10.1016/s0168-8278(85)80779-0 3840503

[46] Kamal S Abdelhakam S Ghoraba D Massoud Y Aziz KA Hassan H . The Frequency, Clinical Course, and Health Related Quality of Life in Adults With Gilbert’s Syndrome: A Longitudinal Study. BMC Gastroenterol (2019) 19(1):22. doi: 10.1186/s12876-019-0931-2 30717703 PMC6360704

[47] Stec DE Hinds TD Jr . Natural Product Heme Oxygenase Inducers as Treatment for Nonalcoholic Fatty Liver Disease. Int J Mol Sci (2020) 21(24):1–16. doi: 10.3390/ijms21249493 PMC7764878 33327438

[48] Toth B Yokoyama Y Kuebler JF Schwacha MG Rue LW 3rd Bland KI . Chaudry IH: Sex Differences in Hepatic Heme Oxygenase Expression and Activity Following Trauma and Hemorrhagic Shock. Arch Surg (2003) 138(12):1375–82. doi: 10.1001/archsurg.138.12.1375 14662543

[49] Sullivan JL . Iron and the Sex Difference in Heart Disease Risk. Lancet (1981) 1(8233):1293–4. doi: 10.1016/s0140-6736(81)92463-6 6112609

[50] Hinds TD Jr. Hosick PA Chen S Tukey RH Hankins MW Nestor-Kalinoski A . Mice With Hyperbilirubinemia Due to Gilbert’s Syndrome Polymorphism Are Resistant to Hepatic Steatosis by Decreased Serine 73 Phosphorylation of PPARalpha. Am J Physiol Endocrinol Metab (2017) 312(4):E244–52. doi: 10.1152/ajpendo.00396.2016 PMC5406988 28096081

[51] Landerer S Kalthoff S Paulusch S Strassburg CP . A Gilbert Syndrome-Associated Haplotype Protects Against Fatty Liver Disease in Humanized Transgenic Mice. Sci Rep (2020) 10(1):8689. doi: 10.1038/s41598-020-65481-4 32457304 PMC7250928

[52] Chalasani N Younossi Z Lavine JE Charlton M Cusi K Rinella M . The Diagnosis and Management of Nonalcoholic Fatty Liver Disease: Practice Guidance From the American Association for the Study of Liver Diseases. Hepatology (2018) 67(1):328–57. doi: 10.1002/hep.29367 28714183

[53] Rinella ME . Nonalcoholic Fatty Liver Disease: A Systematic Review. JAMA (2015) 313(22):2263–73. doi: 10.1001/jama.2015.5370 26057287

[54] Stefano B Fabio N Enrica B Alessandra M Dante R Amedeo L . NAFLD as a Sexual Dimorphic Disease: Role of Gender and Reproductive Status in the Development and Progression of Nonalcoholic Fatty Liver Disease and Inherent Cardiovascular Risk. Adv Ther (2017) 34(6):1291–326. doi: 10.1007/s12325-017-0556-1 PMC5487879 28526997

[55] Yki-Jarvinen H . Non-Alcoholic Fatty Liver Disease as a Cause and a Consequence of Metabolic Syndrome. Lancet Diabetes Endocrinol (2014) 2(11):901–10. doi: 10.1016/S2213-8587(14)70032-4 24731669

[56] Lallukka S Sevastianova K Perttila J Hakkarainen A Orho-Melander M Lundbom N . Adipose Tissue Is Inflamed in NAFLD Due to Obesity but Not in NAFLD Due to Genetic Variation in PNPLA3. Diabetologia (2013) 56(4):886–92. doi: 10.1007/s00125-013-2829-9 23334462

[57] Chen W Tumanov S Fazakerley DJ Cantley J James DE Dunn LL . Bilirubin Deficiency Renders Mice Susceptible to Hepatic Steatosis in the Absence of Insulin Resistance. Redox Biol (2021) 47:102152. doi: 10.1016/j.redox.2021.102152 34610553 PMC8498001

[58] Vitek L Bellarosa C Tiribelli C . Induction of Mild Hyperbilirubinemia: Hype or Real Therapeutic Opportunity? Clin Pharmacol Ther (2019) 106(3):568–75. doi: 10.1002/cpt.1341 30588615

[59] Hinds TD Jr. Creeden JF Gordon DM Stec DF Donald MC Stec DE . Bilirubin Nanoparticles Reduce Diet-Induced Hepatic Steatosis, Improve Fat Utilization, and Increase Plasma Beta-Hydroxybutyrate. Front Pharmacol (2020) 11:594574. doi: 10.3389/fphar.2020.594574 33390979 PMC7775678

[60] McCarty MF . ‘‘iatrogenic Gilbert Syndrome’’–a Strategy for Reducing Vascular and Cancer Risk by Increasing Plasma Unconjugated Bilirubin. Med Hypotheses (2007) 69(5):974–94. doi: 10.1016/j.mehy.2006.12.069 17825497

