# Supplementary material for: Association of Serum Bilirubin With Metabolic Syndrome and Non-Alcoholic Fatty Liver Disease: A Systematic Review and Meta-Analysis
Source: Front Endocrinol (Lausanne). 2022 Jul 20;13:869579. doi: 10.3389/fendo.2022.869579 (PMC9346511; doi:10.3389/fendo.2022.869579)
Supplement: Supplementary file 1 [file DataSheet_1.docx]

# Supplementary Material 1

# MOOSE Checklist for Meta-analyses of Observational Studies

| **Item No** | **Recommendation** | **Reported on Page No** |
| --- | --- | --- |
| Reporting of background should include | | |
| 1 | Problem definition | 3 |
| 2 | Hypothesis statement | 3-4 |
| 3 | Description of study outcome(s) | 4-5 |
| 4 | Type of exposure or intervention used | 5 |
| 5 | Type of study designs used | 5 |
| 6 | Study population | 5 |
| Reporting of search strategy should include | | |
| 7 | Qualifications of searchers (eg, librarians and investigators) | 6 |
| 8 | Search strategy, including time period included in the synthesis and key words | 5 |
| 9 | Effort to include all available studies, including contact with authors | 5-6 |
| 10 | Databases and registries searched | 5 |
| 11 | Search software used, name and version, including special features used (eg, explosion) | 5 |
| 12 | Use of hand searching (eg, reference lists of obtained articles) | 5 |
| 13 | List of citations located and those excluded, including justification | 6 |
| 14 | Method of addressing articles published in languages other than English | 6 |
| 15 | Method of handling abstracts and unpublished studies | 6 |
| 16 | Description of any contact with authors | 6 |
| Reporting of methods should include | | |
| 17 | Description of relevance or appropriateness of studies assembled for assessing the hypothesis to be tested | 6-7 |
| 18 | Rationale for the selection and coding of data (eg, sound clinical principles or convenience) | 6-7 |
| 19 | Documentation of how data were classified and coded (eg, multiple raters, blinding and interrater reliability) | 6 |
| 20 | Assessment of confounding (eg, comparability of cases and controls in studies where appropriate) | 7 |
| 21 | Assessment of study quality, including blinding of quality assessors, stratification or regression on possible predictors of study results | 6 |
| 22 | Assessment of heterogeneity | 7 |
| 23 | Description of statistical methods (eg, complete description of fixed or random effects models, justification of whether the chosen models account for predictors of study results, dose-response models, or cumulative meta-analysis) in sufficient detail to be replicated | 6-7 |
| 24 | Provision of appropriate tables and graphics | 7 |
| Reporting of results should include | | |
| 25 | Graphic summarizing individual study estimates and overall estimate | 7-10 |
| 26 | Table giving descriptive information for each study included | 7-8 |
| 27 | Results of sensitivity testing (eg, subgroup analysis) | 11 |
| 28 | Indication of statistical uncertainty of findings | 11 |

## 1

| **Item No** | **Recommendation** | **Reported on Page**  **No** |
| --- | --- | --- |
| Reporting of discussion should include | | |
| 29 | Quantitative assessment of bias (eg, publication bias) | 12-18 |
| 30 | Justification for exclusion (eg, exclusion of non-English language citations) | 17-18 |
| 31 | Assessment of quality of included studies | 17-18 |
| Reporting of conclusions should include | | |
| 32 | Consideration of alternative explanations for observed results | 18 |
| 33 | Generalization of the conclusions (ie, appropriate for the data presented and within the domain of the literature review) | 18 |
| 34 | Guidelines for future research | 18 |
| 35 | Disclosure of funding source | 19 |

*From*: Stroup DF, Berlin JA, Morton SC, et al, for the Meta-analysis Of Observational Studies in Epidemiology (MOOSE) Group. Meta-analysis of Observational Studies in Epidemiology. A Proposal for Reporting. *JAMA*. 2000;283(15):2008-2012. doi: 10.1001/jama.283.15.2008.

Transcribed from the original paper within the NEUROSURGERY® Editorial Office, Atlanta, GA, United Sates. August 2012.

## 2

### Supplementary Material 2

### Pubmed

search terms

MeSH Terms：Non-alcoholic Fatty Liver Disease Entry Terms：Non alcoholic Fatty Liver Disease NAFLD

Nonalcoholic Fatty Liver Disease Fatty Liver, Nonalcoholic

Fatty Livers, Nonalcoholic Liver, Nonalcoholic Fatty Livers, Nonalcoholic Fatty Nonalcoholic Fatty Liver Nonalcoholic Fatty Livers Nonalcoholic Steatohepatitis Nonalcoholic Steatohepatitides Steatohepatitides, Nonalcoholic Steatohepatitis, Nonalcoholic

MeSH Terms：Metabolic Syndrome Entry Terms：Metabolic Syndromes Syndrome, Metabolic

Syndromes, Metabolic Metabolic Syndrome X

Insulin Resistance Syndrome X Syndrome X, Metabolic Syndrome X, Insulin Resistance Metabolic X Syndrome Syndrome, Metabolic X

X Syndrome, Metabolic Dysmetabolic Syndrome X Syndrome X, Dysmetabolic Reaven Syndrome X Syndrome X, Reaven

Metabolic Cardiovascular Syndrome Cardiovascular Syndrome, Metabolic Cardiovascular Syndromes, Metabolic Syndrome, Metabolic Cardiovascular Cardiometabolic Syndrome Cardiometabolic Syndromes Syndrome, Cardiometabolic Syndromes, Cardiometabolic

MeSH Terms：Bilirubin

Entry Terms：Bilirubin IX alpha Bilirubin, (4E)-Isomer

Bilirubin, (4E,15E)-Isomer Hematoidin

Bilirubin, Disodium Salt Disodium Salt Bilirubin Bilirubin, Monosodium Salt Monosodium Salt Bilirubin delta-Bilirubin

delta Bilirubin Bilirubin, (15E)-Isomer Bilirubin, Calcium Salt Calcium Salt Bilirubin Salt Bilirubin, Calcium Calcium Bilirubinate Bilirubinate, Calcium

.......................................................

1. "Non-alcoholic Fatty Liver Disease"[Mesh]
2. ((((((((((((Non alcoholic Fatty Liver Disease[Title/Abstract]) OR (NAFLD[Title/Abstract])) OR (Nonalcoholic Fatty Liver Disease[Title/Abstract])) OR (Fatty Liver, Nonalcoholic[Title/Abstract])) OR (Fatty Livers, Nonalcoholic[Title/Abstract])) OR (Liver, Nonalcoholic Fatty[Title/Abstract])) OR (Livers, Nonalcoholic Fatty[Title/Abstract])) OR (Nonalcoholic Fatty Liver[Title/Abstract])) OR (Nonalcoholic Fatty Livers[Title/Abstract])) OR (Nonalcoholic Steatohepatitis[Title/Abstract])) OR (Nonalcoholic Steatohepatitides[Title/Abstract])) OR (Steatohepatitides, Nonalcoholic[Title/Abstract])) OR (Steatohepatitis, Nonalcoholic[Title/Abstract])

3. #1 OR #2

1. "Metabolic Syndrome"[Mesh]
2. (((((((((((((((((((((Metabolic Syndromes[Title/Abstract]) OR (Syndrome, Metabolic[Title/Abstract])) OR (Syndromes, Metabolic[Title/Abstract])) OR (Metabolic Syndrome X[Title/Abstract])) OR (Insulin Resistance Syndrome X[Title/Abstract])) OR (Syndrome X, Metabolic[Title/Abstract])) OR (Syndrome X, Insulin Resistance[Title/Abstract])) OR (Metabolic X Syndrome[Title/Abstract])) OR (Syndrome, Metabolic X[Title/Abstract])) OR (X Syndrome, Metabolic[Title/Abstract])) OR (Dysmetabolic Syndrome X[Title/Abstract])) OR (Syndrome X, Dysmetabolic[Title/Abstract])) OR (Reaven Syndrome X[Title/Abstract])) OR (Syndrome X, Reaven[Title/Abstract])) OR (Metabolic Cardiovascular Syndrome[Title/Abstract])) OR (Cardiovascular Syndrome, Metabolic[Title/Abstract])) OR (Cardiovascular Syndromes, Metabolic[Title/Abstract])) OR (Syndrome, Metabolic Cardiovascular[Title/Abstract])) OR (Cardiometabolic Syndrome[Title/Abstract])) OR (Cardiometabolic Syndromes[Title/Abstract])) OR (Syndrome, Cardiometabolic[Title/Abstract])) OR (Syndromes, Cardiometabolic[Title/Abstract])

6. #4 OR #5

1. "Bilirubin"[Mesh]
2. (((((((((((((((Bilirubin IX alpha[Title/Abstract]) OR (Bilirubin, (4E)-Isomer[Title/Abstract])) OR (Bilirubin, (4E,15E)-Isomer[Title/Abstract])) OR (Hematoidin[Title/Abstract])) OR (Bilirubin,

Disodium Salt[Title/Abstract])) OR (Disodium Salt Bilirubin[Title/Abstract])) OR (Bilirubin, Monosodium Salt[Title/Abstract])) OR (Monosodium Salt Bilirubin[Title/Abstract])) OR (delta-Bilirubin[Title/Abstract])) OR (delta Bilirubin[Title/Abstract])) OR (Bilirubin, (15E)-Isomer[Title/Abstract])) OR (Bilirubin, Calcium Salt[Title/Abstract])) OR (Calcium Salt Bilirubin[Title/Abstract])) OR (Salt Bilirubin, Calcium[Title/Abstract])) OR (Calcium Bilirubinate[Title/Abstract])) OR (Bilirubinate, Calcium[Title/Abstract])

9. #7 OR #8

10. #3 AND #9

11. #6 AND #9 12. #10 OR #11

### Cochrane Library

search terms

MeSH Terms：Non-alcoholic Fatty Liver Disease Entry Terms：Steatohepatitides, Nonalcoholic; Nonalcoholic Steatohepatitides;

Steatohepatitis, Nonalcoholic; Nonalcoholic Steatohepatitis; Nonalcoholic Fatty Liver; Nonalcoholic Fatty Livers;

Non alcoholic Fatty Liver Disease; Livers, Nonalcoholic Fatty;

Liver, Nonalcoholic Fatty;

Fatty Livers, Nonalcoholic;

Fatty Liver, Nonalcoholic;

NAFLD;

Nonalcoholic Fatty Liver Disease

MeSH Terms：Metabolic Syndrome

Entry Terms：Syndrome X, Insulin Resistance; Metabolic Syndromes;

Syndrome, Metabolic Cardiovascular; Metabolic Cardiovascular Syndrome; Dysmetabolic Syndrome X; Syndrome, Metabolic;

Reaven Syndrome X; Syndrome X, Reaven; Syndromes, Metabolic;

Insulin Resistance Syndrome X; Cardiovascular Syndrome, Metabolic; Syndrome, Metabolic X; Cardiovascular Syndromes, Metabolic;

Syndrome X, Dysmetabolic; Syndrome X, Metabolic; Metabolic X Syndrome;

X Syndrome, Metabolic; Metabolic Syndrome X; Cardiometabolic Syndrome; Syndrome, Cardiometabolic; Cardiometabolic Syndromes; Syndromes, Cardiometabolic

MeSH Terms：Bilirubin

Entry Terms：Bilirubin, (4E)-Isomer; Hematoidin;

Bilirubin, Disodium Salt; Disodium Salt Bilirubin; Bilirubin, (4E,15E)-Isomer; Bilirubin IX alpha; Bilirubin, (15E)-Isomer; Monosodium Salt Bilirubin; Bilirubin, Monosodium Salt; Bilirubinate, Calcium; Calcium Bilirubinate; Bilirubin, Calcium Salt;

Salt Bilirubin, Calcium; Calcium Salt Bilirubin; delta Bilirubin;

delta-Bilirubin

.......................................................

#1: MeSH descriptor: [Non-alcoholic Fatty Liver Disease] explode all trees

#2: (Steatohepatitides, Nonalcoholic):ti,ab,kw OR (Nonalcoholic Steatohepatitides):ti,ab,kw OR (Steatohepatitis, Nonalcoholic):ti,ab,kw OR (Nonalcoholic Steatohepatitis):ti,ab,kw OR (Nonalcoholic Fatty Liver):ti,ab,kw

#3: (Nonalcoholic Fatty Livers):ti,ab,kw OR (Non alcoholic Fatty Liver Disease):ti,ab,kw OR (Livers, Nonalcoholic Fatty):ti,ab,kw OR (Liver, Nonalcoholic Fatty):ti,ab,kw OR (Fatty Livers,

Nonalcoholic):ti,ab,kw

#4: (Fatty Liver, Nonalcoholic):ti,ab,kw OR (NAFLD):ti,ab,kw OR (Nonalcoholic Fatty Liver Disease):ti,ab,kw

#5: #1 OR #2 OR #3 OR #4

#6: MeSH descriptor: [Metabolic Syndrome] explode all trees

#7: (Syndrome X, Insulin Resistance):ti,ab,kw OR (Metabolic Syndromes):ti,ab,kw OR (Syndrome, Metabolic Cardiovascular):ti,ab,kw OR (Metabolic Cardiovascular Syndrome):ti,ab,kw OR (Dysmetabolic Syndrome X):ti,ab,kw

#8: (Syndrome, Metabolic):ti,ab,kw OR (Reaven Syndrome X):ti,ab,kw OR (Syndrome X,

Reaven):ti,ab,kw OR (Syndromes, Metabolic):ti,ab,kw OR (Insulin Resistance Syndrome X):ti,ab,kw

#9: (Cardiovascular Syndrome, Metabolic):ti,ab,kw OR (Syndrome, Metabolic X):ti,ab,kw OR (Cardiovascular Syndromes, Metabolic):ti,ab,kw OR (Syndrome X, Dysmetabolic):ti,ab,kw OR (Syndrome X, Metabolic):ti,ab,kw

#10: (Metabolic X Syndrome):ti,ab,kw OR (X Syndrome, Metabolic):ti,ab,kw OR (Metabolic Syndrome X):ti,ab,kw OR (Cardiometabolic Syndrome):ti,ab,kw OR (Syndrome,

Cardiometabolic):ti,ab,kw

#11: (Cardiometabolic Syndromes):ti,ab,kw OR (Syndromes, Cardiometabolic):ti,ab,kw #12: #6 OR #7 OR #8 OR #9 OR #10 OR #11

#13: MeSH descriptor: [Bilirubin] explode all trees

#14: (Bilirubin, Isomer):ti,ab,kw OR (Hematoidin):ti,ab,kw OR (Bilirubin, Disodium Salt):ti,ab,kw OR (Disodium Salt Bilirubin):ti,ab,kw OR (Bilirubin IX alpha):ti,ab,kw

#15: (Monosodium Salt Bilirubin):ti,ab,kw OR (Bilirubin, Monosodium Salt):ti,ab,kw OR (Bilirubinate, Calcium):ti,ab,kw OR (Calcium Bilirubinate):ti,ab,kw OR (Bilirubin, Calcium Salt):ti,ab,kw

#16: (Salt Bilirubin, Calcium):ti,ab,kw OR (Calcium Salt Bilirubin):ti,ab,kw OR (delta Bilirubin):ti,ab,kw OR (delta-Bilirubin):ti,ab,kw

#17: #13 OR #14 OR #15 OR #16

#18: #5 AND #17

#19: #12 AND #17

#20: #18 OR #19

### Embase

search terms

MeSH Terms：nonalcoholic fatty liver

Entry Terms：NAFLD (nonalcoholic fatty liver disease); non alcoholic fatty liver disease;

non alcoholic hepato-steatosis; non alcoholic hepatosteatosis; non alcoholic liver steatosis;

non alcoholic steatotic hepatopathy; non-alcoholic fatty liver;

non-alcoholic fatty liver disease; non-alcoholic FLD;

non-alcoholic hepatic steatosis; nonalcoholic fatty liver disease; nonalcoholic FLD; nonalcoholic hepatic steatosis; nonalcoholic hepatosteatosis; nonalcoholic liver steatosis

MeSH Terms：metabolic syndrome X

Entry Terms：insulin resistance syndrome; metabolic syndrome;

syndrome X, metabolic

MeSH Terms：bilirubin

Entry Terms：1, 3, 6, 7 tetramethyl 4, 5 dicarboxyethyl 2, 8 divinyl (b 13) dihydrobilenone; bilirubin acid;

bilirubin delta; bilirubin ix alpha; bilirubin ixalpha; bilirubin pigment; bilirubin sulfate isomer;

bilirubin sulphate isomer; bilirubinate;

bilirubine; bilirubinoid; calcium bilirubinate;

haematoidin; hematoidin; indirect bilirubin; mesobilirubin; unconjugated bilirubin

....................................................... #12. #10 OR #11

#11. #6 AND #9

#10. #3 AND #6

#9. #7 OR #8

#8. 'insulin resistance syndrome;':ab,ti OR 'metabolic syndrome;':ab,ti OR 'syndrome x, metabolic':ab,ti

#7. 'metabolic syndrome x'/exp #6. #4 OR #5

#5. '1, 3, 6, 7 tetramethyl 4, 5 dicarboxyethyl 2, 8 divinyl':ab,ti AND 'b 13':ab,ti AND dihydrobilenone;:ab,ti OR 'bilirubin acid;':ab,ti OR 'bilirubin delta;':ab,ti OR 'bilirubin ix alpha;':ab,ti OR 'bilirubin ixalpha;':ab,ti OR 'bilirubin pigment;':ab,ti OR 'bilirubin sulfate isomer;':ab,ti OR 'bilirubin sulphate isomer;':ab,ti OR bilirubinate;:ab,ti OR bilirubine;:ab,ti OR bilirubinoid;:ab,ti OR 'calcium bilirubinate;':ab,ti OR 'haematoidin; hematoidin;':ab,ti OR 'indirect bilirubin;':ab,ti OR mesobilirubin;:ab,ti OR 'unconjugated bilirubin':ab,ti

#4. 'bilirubin'/exp

#3. #1 OR #2

#2. nafld:ab,ti AND 'nonalcoholic fatty liver disease':ab,ti AND ;:ab,ti OR 'non alcoholic fatty liver disease;':ab,ti OR 'non alcoholic hepato-steatosis;':ab,ti OR 'non alcoholic hepatosteatosis;':ab,ti OR 'non alcoholic liver steatosis;':ab,ti OR 'non alcoholic steatotic hepatopathy;':ab,ti OR 'non-alcoholic fatty liver;':ab,ti OR 'non-alcoholic fatty liver disease;':ab,ti OR 'non-alcoholic fld;':ab,ti OR 'non-alcoholic hepatic steatosis;':ab,ti OR 'nonalcoholic fatty liver

disease;':ab,ti OR 'nonalcoholic fld;':ab,ti OR 'nonalcoholic hepatic steatosis;':ab,ti OR 'nonalcoholic hepatosteatosis;':ab,ti OR 'nonalcoholic liver steatosis':ab,ti

#1. 'nonalcoholic fatty liver'/exp

### Supplementary Material 3

# Publication Bias


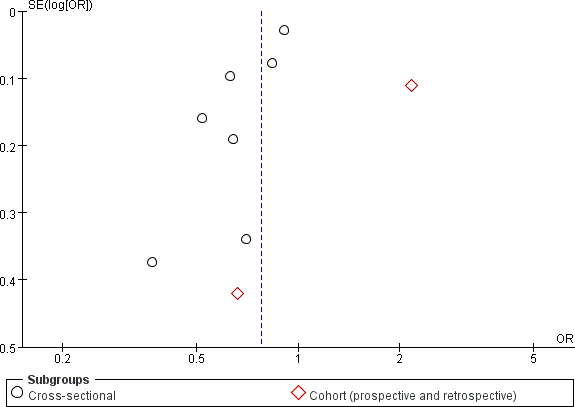


**Supplementary Figure 1.** Funnel plot for association of metabolic syndrome (MetS) with total bilirubin (TBIL) among female group


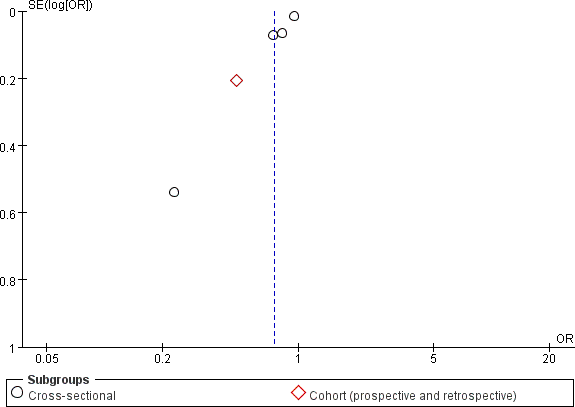


**Supplementary Figure 2.** Funnel plot for association of metabolic syndrome (MetS) with total

bilirubin (TBIL) among gender-neutral group.


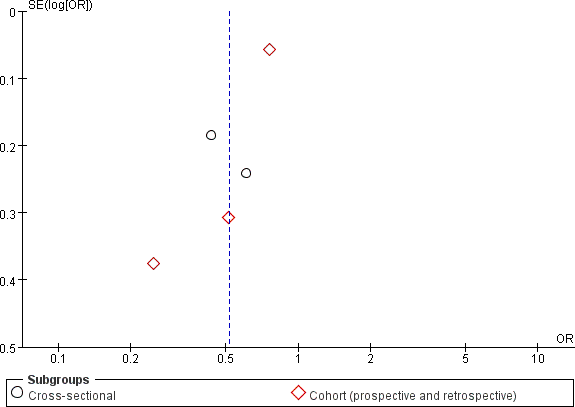


**Supplementary Figure 3.** Funnel plot for association of metabolic syndrome (MetS) with direct bilirubin (DBIL) among male group


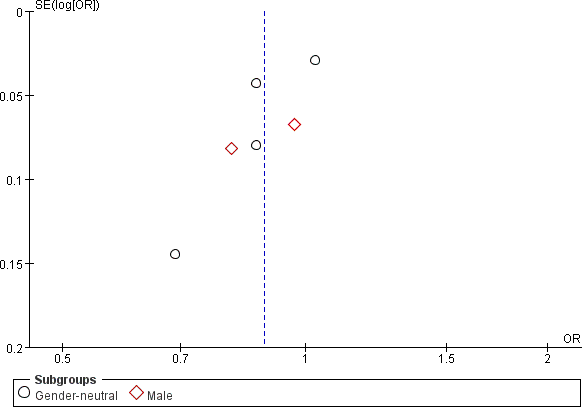


**Supplementary Figure 4.** Funnel plot for association of non-alcoholic fatty liver disease (NAFLD) with total bilirubin (TBIL)


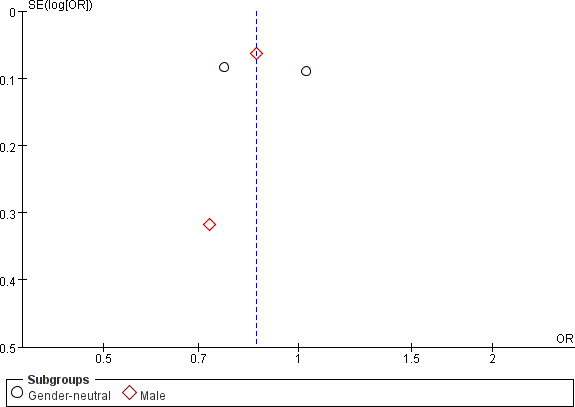


**Supplementary Figure 5.** Funnel plot for association of non-alcoholic fatty liver disease (NAFLD) with direct bilirubin (DBIL)
